# The dataset for validation of factors affecting pre-service teachers' use of ICT during teaching practices: Indonesian context

**DOI:** 10.1016/j.dib.2019.104875

**Published:** 2019-11-26

**Authors:** Akhmad Habibi, Farrah Dina Yusop, Rafiza Abdul Razak

**Affiliations:** a Department of Curriculum & Instructional Technology, Faculty of Education, University of Malaya, Malaysia; b Universitas Jambi, Indonesia

**Keywords:** TPACK, Beliefs on ICT, Use of ICT, Pre-service teachers, Validity and reliability

## Abstract

This dataset describes two main variables, technological pedagogical and content knowledge (TPACK) and Beliefs on ICT, which may affect pre-service teachers' (PSTs) use of ICT (UICT) during teaching practices. TPACK assumes that PSTs should actively combine some domains of knowledge to design good quality of ICT-integrated courses lessons. Beliefs on ICT in this study consist of a mix of behavioral, normative, and control beliefs on ICT integration in education. In addition, UICT is defined as ICT used by PSTs during teaching practices. Three approaches were applied for the purification of the dataset; development of instruments, survey, and exploratory factor analysis (EFA). The dataset consists of demographic information, TPACK, Beliefs on ICT, and UICT. The dataset is beneficial to teacher educators in designing effective programs that best nurture PSTs' UICT during teaching practices. Researchers sharing similar sample characteristics in developing countries may adapt this dataset for more rigorous statistical analyses.

**Table:** Specifications Table

Subject area	Education
More specific subject area	Educational technology
Type of data	Tables
How data was acquired	Face and content validity, survey, and EFA
Data format	Raw, analyzed
Experimental factors	Demographic information, TPACK, Beliefs on ICT, UICT
Experimental features	Raw data was adapted from previous studies and validated through face and content validity before it was distributed to the respondents. The responses were analyzed through EFA
Data source location	Data gathered from three universities in Jambi, Indonesia
Data accessibility	Raw and analyzed data were deposited at Mendeley Data, v1 https://data.mendeley.com/datasets/s6brgxxktt/7
Related research article	Factors affecting Indonesian pre-service teachers' integration of ICT during teaching practices (In press) [1]

**Table:** 

**Value of the Data** • The dataset informs an insight into factors affecting UICT during teaching practices for Indonesian contexts. • The dataset is beneficial to teacher educators in designing effective programs that best nurture PSTs' UICT during teaching practices. • Open access of this dataset has a potential ability to be adapted by related stakeholders to gain an understanding of the practices of technology into education in pre-service teacher training programs. • Researchers sharing similar sample characteristics in developing countries may adapt this dataset for more rigorous statistical analyses.

## 1 Data

**Fig. 1:**
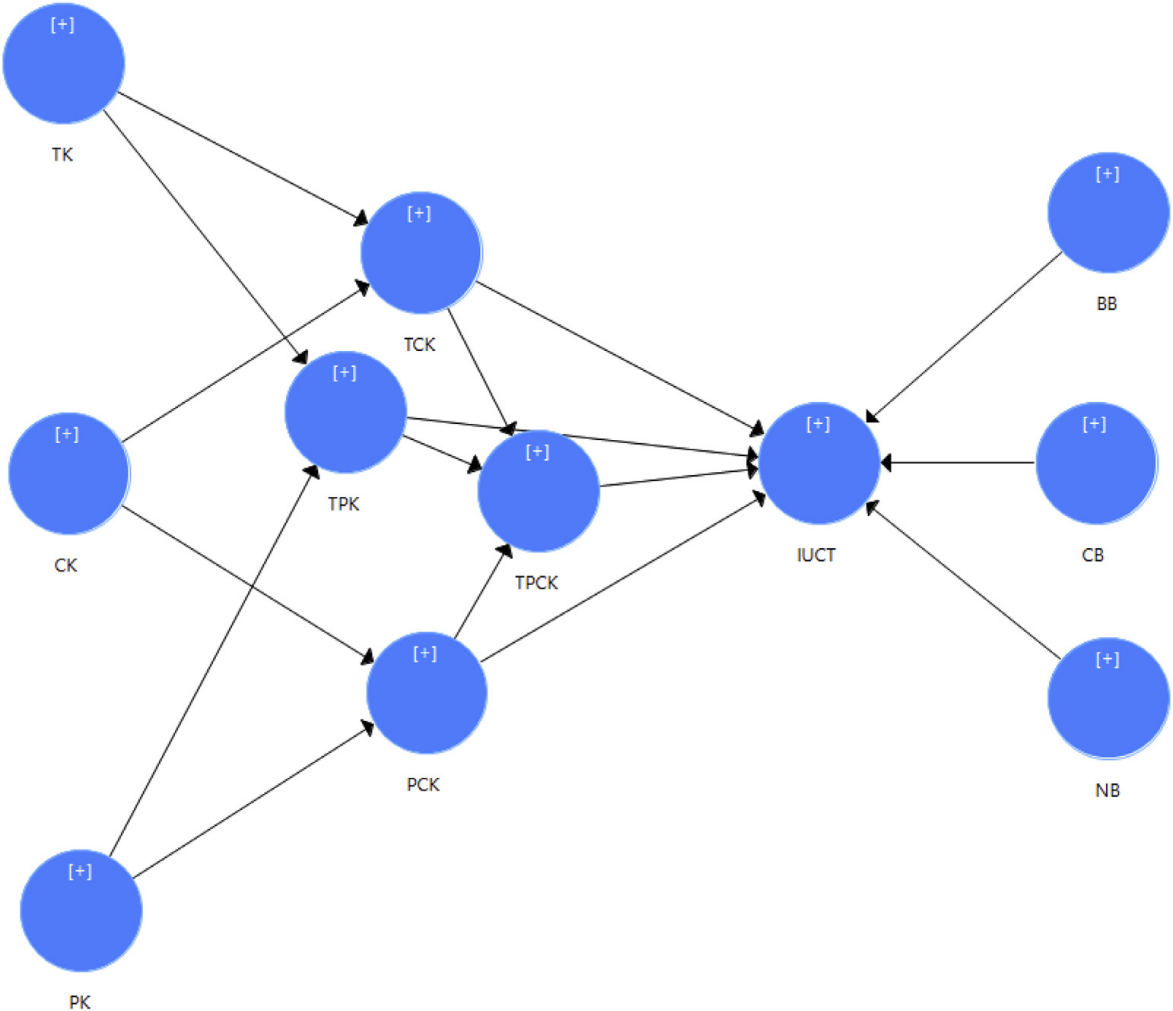
Study model.

This dataset contains variables' dimension, definition, and adapted references of the instruments (Table 1) as well as versions of the instruments during the development process (Table 2). Further, the instruments were distributed as a survey to PSTs from three Indonesian universities. For normality test, Skewness and Kurtosis were calculated for 3 variables (TPACK, Beliefs on ICT, and UICT). The reliability was also examined by calculating the Cronbach's alpha (Table 3). Finally, EFA was conducted by examining two variables' (TPACK and Beliefs on ICT) Kaiser-Meyer-Olkin (KMO), Bartlett's Test of Sphericity, Eigenvalue, and cross loading (Table 4, Table 5, Table 6, Table 7). The raw and analyzed data were accessible at Mendeley Data, https://data.mendeley.com/datasets/s6brgxxktt/7. Fig. 1 exhibits the study model.

**Table 1:** Variables' dimension, definition, and adapted references of the survey instruments.

Variable	Dimension	Definition	Adapted references of the survey instrument
TPACK	Technological knowledge (TK)	Knowledge of emerging technologies for ICT integration during teaching practices	[2]
	Content knowledge (CK)	Knowledge of teaching such as teaching principles, students' psychology of students, teaching strategies, and management of class during teaching practices	[7,8]
	Pedagogical knowledge (PK)	Subject matter knowledge e.g. scientific, social, and linguistics knowledge during teaching practices	[2]
	Pedagogical and content knowledge (PCK)	Knowledge of changing specific content into an understandable and accessible form for learners via an approach of pedagogy during teaching practices	[2]
	Technological content knowledge (TCK)	Knowledge of integrating emerging technologies for certain subject matter knowledge which excludes pedagogical aims during teaching practices	[2]
	Technological pedagogical knowledge (TPK)	Knowledge of integrating emerging technologies in pedagogy during teaching practices	[7,8]
	Technological pedagogical content knowledge (TPCK)	Knowledge of implementing technologies to improve students' understanding and learning in certain subject matter knowledge during teaching practices	[2,8]
Beliefs on ICT	Behavioral beliefs (BB)	Associated with attitudes for integrating ICT during teaching practices/Outcomes of using ICT	[9,10]
	Normative beliefs (NB)	Associated with subjective norms for integrating ICT during teaching practices/People who expect the use of ICT	[9,10]
	Control beliefs (CB)	Associated with perceived behavioral control/Internal and external enablers/constraints	[9,10]
UICT	UICT	UICT during teaching practices reflected on their integration evaluation it during actual placement	[4]

**Table 2:** Versions of the instruments during the development process.

Variable	Version 1 Phase 1 (adaptation, construction, and translation)		Version 2 Phase 2 (face and content validity; discussion with 5 users and 5 experts)		Version 3 Phase 2 (face and content validity; CVI with 10 experts)	
	Dimension	Number of items	Dimension	Number of items	Dimension	Number of items
TPACK	TK	7	TK	3	TK	3
	CK	3	CK	3	CK	3
	PK	7	PK	7	PK	7
	PCK	3	PCK	3	PCK	3
	TCK	4	TCK	3	TCK	3
	TPK	4	TPK	4	TPK	4
	TPACK	10	TPACK	5	TPACK	5
Beliefs on ICT	BB	9	BB	8	BB	8
	NB	7	NB	5	NB	5
	CB	7	Cb	5	CB	5
UICT	UICT	11	UICT	12	UICT	12
Total		70		58		58

**Table 3:** Skewness, Kurtosis, and Cronbach's alpha of TPACK, Beliefs on ICT, and UICT.

	N	Skewness		Kurtosis		Reliability
	Statistic	Statistic	Std. Error	Statistic	Std. Error	α
TK	287	–.275	.144	1.012	.287	.829
CK	287	–.382	.144	1.217	.287	.749
PK	287	–.044	.144	1.098	.287	.867
PCK	287	–.392	.144	1.735	.287	.871
TCK	287	–.111	.144	1.494	.287	.841
TPK	287	–.343	.144	.226	.287	.766
TPACK	287	–.175	.144	1.056	.287	.845
BB	287	–.202	.144	.043	.287	.884
NB	287	–.144	.144	.563	.287	.843
CB	287	–.185	.144	1.370	.287	.849
UICT	287	.082	.144	.267	.287	.895

**Table 4:** KMO and Bartlett's test of TPACK.

KMO		.901
Bartlett's Test of Sphericity	Approx. Chi-Square	3898.011
	df	325
	Sig.	p < .001

**Table 5:** Eigenvalue and cross loading of TPACK.

Construct	Eigenvalue		Items	Component						
	Total	% of Variance		1	2	3	4	5	6	7
PK	9.296	35.754	PK5	.805						
			PK7	.752						
			PK1	.693						
			PK6	.653						
			PK3	.579						
			PK2	.530						
TPACK	2.378	9.146	TPACK2		.764					
			TPACK4		.741					
			TPACK1		.685					
			TPACK5		.683					
			TPACK3		.539					
PCK	1.550	5.962	PCK2			.849				
			PCK3			.842				
			PCK1			.746				
CK	1.427	5.488	CK3				.762			
			CK2				.730			
			CK1				.504			
TCK	1.312	5.044	TCK2					.847		
			TCK3					.800		
			TCK1					.732		
TK	1.264	4.860	TK2						.835	
			TK3						.775	
			TK1						.748	
TPK	1.012	3.890	TPK3							.847
			TPK4							.810
			TPK2							.513

**Table 6:** KMO and Bartlett's test of Beliefs on ICT.

KMO		.915
Bartlett's Test of Sphericity	Approx. Chi-Square	2501.441
	df	136
	Sig.	p < .001

**Table 7:** Eigenvalue and cross loading of Beliefs on ICT.

Construct	Eigenvalue		Item	Component		
	Total	% of Variance		1	2	3
BB	7.577	44.569	BB4	.821		
			BB5	.747		
			BB3	.740		
			BB2	.682		
			BB7	.625		
			BB6	.617		
			BB8	.562		
CB	1.585	9.325	CB4		.830	
			CB3		.742	
			CB5		.693	
			CB1		.668	
			CB2		.639	
NB	1.203	7.074	NB2			.772
			NB1			.709
			NB3			.701
			NB4			.694
			NB5			.663

## 2 Experimental design, materials, and methods

TPACK is the framework assuming that PSTs should actively combine some domains of knowledge to design good quality of ICT-integrated courses lessons [2]. Beliefs on ICT in this study consist of a mix of behavioral, normative, and control beliefs on ICT integration in education [3]. In addition, UICT is defined as ICT used by PSTs during teaching practices [4]. Three approaches were applied for the purification of the dataset; development of the instruments, survey, and EFA.

### 2.1 Development of the instruments

For the development of the instruments, a 2-phase strategy introduced by Ref. [5] was applied. In the 1st phase, the processes of adaptation, construction, and translation were conducted. The adaptation, construction, and translation process include demographic information, TPACK, Beliefs on ICT, and UICT. Demographic information consists of questions about, age, major, university, and ICT-based courses. Table 1 exhibits the variables' dimension, definition, and adapted references in the adaptation and construction process. Besides, the instruments were translated using the back-translation method [6] English to Indonesian and Indonesia to English, involving 2 translation experts.

In the 2nd phase, face and content validity were done. Firstly, two panels of 5 users and 5 experts were involved in 2 discussion sessions to evaluate the instruments for their context and setting appropriateness. Further, the instruments were distributed to 10 experts of educational technology who agree to participate to examine the instruments' relevance, clarity, and simplicity as part of the content validity index (CVI). The attributes of the instruments' items were rated on a 4-point scale 1 = not relevant/not clear/not simple to 4 = very relevant/very clear/very simple [5]. The CVI was measured at the item level (I-CVI) for three variables (TPACK, Beliefs on ICT, and UICT). The I-CVI was measured by providing a score of 3 or 4 divided by the total number of experts [5]. With a total of ten experts, the I-CVI should not be less than 0.78 [11]. Additionally, a modified Kappa (k*) index was calculated to have an estimation to the I-CVI [11]. The k* is an index of agreement from the experts indicating that the item is relevant, clear, simple. To calculate k*, the probability of chance occurrence (Pc) was first calculated [11]. The standards recommended by Ref. [12] were adopted to interpret k* in which the values above 0.74, between 0.60 and 0.74, and between 0.40 and 0.59 are defined as excellent, good, and fair, respectively. The calculation and information of CVI, k*, and Pc can be accessed at the Mendeley website as informed earlier.

### 2.2 Survey

After the development process, the instruments were distributed as a form of a survey to PSTs from three Indonesian universities. A survey design was chosen because it elaborates trends of the data rather than inform rigorous explanations. The total population of the study was all Indonesian PSTs while the target population is PSTs in the three universities. The sample was determined through simple random sampling. The instruments were distributed to 300 PSTs in which 287 responses were measurable; 10 of them were not completed and 3 were not returned.

Data normality was assessed by calculating Skewness and Kurtosis. Skewness and kurtosis values need to be in the range of −2 to +2 [13]. All Skewness and Kurtosis values are within the recommended range values (Table 3). In addition, the reliability of data was conducted through Cronbach's alpha (α > 0.700). Table 3 performs all value of Cronbach's alpha and no values are less than 0.700.

### 2.3 Exploratory factor analysis (EFA)

EFA was conducted for TPACK and Beliefs on ICT as two main variables of factors that may affect UICT during teaching practices (Fig. 1). UICT was not included in this process since it was theoretically defined as one factor. A three-time rotation of factor analysis was run in SPSS 23 that included 28 items for TPACK and 18 items for Beliefs on ICT. Both TPACK and Beliefs on ICT data were analyzed through principal component analysis with Varimax rotation for four assessments; KMO, Bartlett's Test of Sphericity, Eigenvalue, and cross loading. The value of KMO for TPACK is 0.901 with Bartlett's Test of Sphericity value was significant (*p* < .001). In addition, the value of KMO for Beliefs on ICT is 0.915 and the value of Bartlett's Test of Sphericity was also significant (*p* < .001). Therefore, no issues are indicated for KMO and Bartlett's Test of Sphericity of TPACK and Beliefs on ICT (Table 3, Table 6). From the rotation, 7 factors were extracted and labeled according to the theories of TPACK. The eigenvalue of the seven factors ranged from 9.296 to 1.012 with a maximal percentage of the variance of 35.754% (Table 5). Through the process, TPK 1 and PK 4 were dropped because the cross loading values of the items were highly detected. For Beliefs on ICT, 3 factors were extracted and were labeled behavioral, normative, and control belief (Table 7). The eigenvalue of the three factors ranged from 7.577 to 1.203 with the maximal percentage of the variance of 44.569%. No items of Beliefs on ICT were dropped from this process.

## 3 Conclusion

Many frameworks have been established to measure factors affecting technology integration in education such as technology acceptance model, theory of planned behavior, unified theory of acceptance and use of technology, and TPACK. However, little research has informed UICT predicted by combined frameworks. Therefore, this study model is offered as a combination of two frameworks, TPACK and Beliefs on ICT, as factors that may affect UICT. The model is relevant to the current condition of Indonesia as a developing country where technology is massively used in education. The dataset informs an insight into factors affecting UICT during teaching practices for Indonesian contexts. It is expected to be beneficial to teacher educators in designing effective programs that best nurture PSTs' UICT during teaching practices. It also benefits researchers sharing similar sample characteristics in developing countries to adapt this dataset.
