# Supplementary material for: The dataset for validation of factors affecting pre-service teachers' use of ICT during teaching practices: Indonesian context
Source: Data Brief. 2019 Nov 26;28:104875. doi: 10.1016/j.dib.2019.104875 (PMC6911973; doi:10.1016/j.dib.2019.104875)
Supplement: Multimedia component 1 [file mmc1.zip › Content validity index process.docx]

TPACK (CVI process)

| Construct (code) | No of items | Items |
| --- | --- | --- |
| Technological knowledge (TK) | 3 | TK1, TK2, TK3 |
| Content knowledge (CK) | 3 | CK1, CK2, CK3 |
| Pedagogical Knowledge (PK) | 7 | PK1, PK2, PK3, PK4, PK5, PK6, PK7 |
| Technological Pedagogical Knowledge (TCK) | 3 | TCK1, TCK2, TCK3 |
| Pedagogical Content Knowledge (PCK) | 3 | PCK1, PCK2, PCK3 |
| Technological Pedagogical Knowledge (TPK) | 4 | TPK1, TPK2, TPK3, TPK4 |
| Technological Pedagogical and Content Knowledge (TPACK) | 5 | TPACK1, TPACK2, TPACK3, TPACK4, TPACK5 |

Beliefs on ICT (CVI process)

| Construct (code) | No of items | items |
| --- | --- | --- |
| Behavioral beliefs (BB) | 8 | BB1, BB2, BB3, BB4, BB5, BB6, BB7, BB8 |
| Normative beliefs (NB) | 5 | NB1, NB2, NB3, NB4, NB5 |
| Control beliefs (CB) | 5 | CB1, CB2, CB3, CB4, CB5 |

Pre-service teachers ICT integration during teaching practices

| Construct (code) | No of items | items |
| --- | --- | --- |
| Pre-service integration of ICT during teaching practices (UICT) | 12 | UICT1, UICT2, UICT3, UICT4, UICT5, UICT6, UICT7, UICT8, UICT9, UICT10, UICT11, and UICT12. |

Table 4.1

Content validity of the dimension “Technological Knowledge”

| Item | Number  of experts | Relevance of the questions | | | | | Clarity of the questions | | | | | Simplicity of the questions | | | | |
| --- | --- | --- | --- | --- | --- | --- | --- | --- | --- | --- | --- | --- | --- | --- | --- | --- |
|  |  | Number of ratings  (3 or 4) | I-CVI | P_C_ | k* | Evalu  Ation | Number  of ratings (3 or 4) | I-CVI | Pc | k* | Evalu  ation | Number of ratings (3 or 4) | I-CVI | Pc | k* | Evalu  Ation |
| 1 | 10 | 10 | 1.00 | 0.001 | 1.00 | **** | 10 | 1.00 | 0.001 | 1.00 | **** | 9 | 0.90 | 0.010 | 0.90 | **** |
| 2 | 10 | 8 | 0.80 | 0.044 | 0.79 | **** | 8 | 0.80 | 0.044 | 0.79 | **** | 10 | 1.00 | 0.001 | 1.00 | **** |
| 3 | 10 | 9 | 0.90 | 0.010 | 0.90 | **** | 10 | 1.00 | 0.001 | 1.00 | **** | 10 | 1.00 | 0.001 | 1.00 | **** |

I-CVI (content validity index) = number of experts providing a rating of 3 or 4/number of experts
Pc (probability of chance occurrence) = [N!/A!(N-A)!] × 0.5N, N = number of experts; A = number of experts agreeing on a rating of 3 or 4
k* (modified kappa) = (I-CVI-pc)(1-pc)
Evaluation criteria for level of content validity: relationship between I-CVI and k*; excellent validity = I-CVI ≥ 0.78 and k* >0.74 (****); good validity I-CVI < 0.78 and ≥ 0.60 and k* ≤0.74 (***); fair validity I-CVI < 0.6 and ≥ 0.40 and k* ≤0.59 (**); poor validity I-CVI < 0.4 and k* <0.40 (*)

Table 4.2

Content validity of the dimension “Content Knowledge”

|  |  | Relevance of the questions | | | | | Clarity of the questions | | | | | Simplicity of the questions | | | | |
| --- | --- | --- | --- | --- | --- | --- | --- | --- | --- | --- | --- | --- | --- | --- | --- | --- |
| Item | Number  of experts | Number of  ratings | I-CVI | P_C_ | k* | Evalu  Ation | Number  of ratings | I-CVI | Pc | k* | Evalu  ation | Number of  ratings | I-CVI | Pc | k* | Evalu  ation |
| 1 | 10 | 10 | 1.00 | 0.001 | 1.00 | **** | 10 | 1.00 | 0.001 | 1.00 | **** | 10 | 1.00 | 0.001 | 1.00 | **** |
| 2 | 10 | 9 | 0.90 | 0.010 | 0.90 | **** | 10 | 1.00 | 0.001 | 1.00 | **** | 9 | 0.90 | 0.010 | 0.90 | **** |
| 3 | 10 | 9 | 0.90 | 0.010 | 0.90 | **** | 10 | 1.00 | 0.001 | 1.00 | **** | 10 | 1.00 | 0.001 | 1.00 | **** |
| 4 | 10 | 10 | 1.00 | 0.001 | 1.00 | **** | 9 | 0.90 | 0.010 | 0.90 | **** | 10 | 1.00 | 0.001 | 1.00 | **** |

Table 4.2

Content validity of the dimension “Pedagogical Knowledge”

|  |  | Relevance of the questions | | | | | Clarity of the questions | | | | | Simplicity of the questions | | | | |
| --- | --- | --- | --- | --- | --- | --- | --- | --- | --- | --- | --- | --- | --- | --- | --- | --- |
| Item | Number  of experts | Number of  Ratings (3 or 4) | I-CVI | P_C_ | k* | Evalu  Ation | Number  of ratings (3 or 4) | I-CVI | Pc | k* | Evalu  ation | Number of  Ratings (3 or 4) | I-CVI | Pc | k* | Evalu  ation |
| 1 | 10 | 10 | 1.00 | 0.001 | 1.00 | **** | 10 | 1.00 | 0.001 | 1.00 | **** | 10 | 1.00 | 0.001 | 1.00 | **** |
| 2 | 10 | 10 | 1.00 | 0.001 | 1.00 | **** | 10 | 1.00 | 0.001 | 1.00 | **** | 9 | 0.90 | 0.010 | 0.90 | **** |
| 3 | 10 | 9 | 0.90 | 0.010 | 0.90 | **** | 10 | 1.00 | 0.001 | 1.00 | **** | 10 | 1.00 | 0.001 | 1.00 | **** |
| 4 | 10 | 10 | 1.00 | 0.001 | 1.00 | **** | 10 | 1.00 | 0.001 | 1.00 | **** | 9 | 0.90 | 0.010 | 0.90 | **** |
| 5 | 10 | 9 | 0.90 | 0.010 | 0.90 | **** | 10 | 1.00 | 0.001 | 1.00 | **** | 10 | 1.00 | 0.001 | 1.00 | **** |
| 6 | 10 | 10 | 1.00 | 0.001 | 1.00 | **** | 10 | 1.00 | 0.001 | 1.00 | **** | 8 | 0.80 | 0.044 | 0.79 | **** |
| 7 | 10 | 10 | 1.00 | 0.001 | 1.00 | **** | 10 | 1.00 | 0.001 | 1.00 | **** | 10 | 1.00 | 0.001 | 1.00 | **** |

Table 4.2

Content validity of the dimension “Pedagogical Content Knowlegde”

|  |  | Relevance of the questions | | | | | Clarity of the questions | | | | | Simplicity of the questions | | | | |
| --- | --- | --- | --- | --- | --- | --- | --- | --- | --- | --- | --- | --- | --- | --- | --- | --- |
| Item | Number  of experts | Number of ratings (3 or 4) | I-CVI | P_C_ | k* | Evalu  Ation | Number  of ratings (3 or 4) | I-CVI | Pc | k* | Evalu  ation | Number of ratings (3 or 4) | I-CVI | Pc | k* | Evalu  ation |
| 1 | 10 | 10 | 1.00 | 0.001 | 1.00 | **** | 10 | 1.00 | 0.001 | 1.00 | **** | 10 | 1.00 | 0.001 | 1.00 | **** |

Table 4.2

Content validity of the dimension “Technological Content Knowlegde

|  |  | Relevance of the questions | | | | | Clarity of the questions | | | | | Simplicity of the questions | | | | |
| --- | --- | --- | --- | --- | --- | --- | --- | --- | --- | --- | --- | --- | --- | --- | --- | --- |
| Item | Number  of experts | Number of  Ratings (3 or 4) | I-CVI | P_C_ | k* | Evalu  ation | Number  of ratings (3 or 4) | I-CVI | Pc | k* | Evalu  ation | Number of  Ratings (3 or 4) | I-CVI | Pc | k* | Evalu  ation |
| 1 | 10 | 10 | 1.00 | 0.001 | 1.00 | **** | 10 | 1.00 | 0.001 | 1.00 | **** | 9 | 1.00 | 0.001 | 1.00 | **** |

Table 4.2

Content validity of the dimension “Technological Pedagogical Knowledge”

|  |  | Relevance of the questions | | | | | Clarity of the questions | | | | | Simplicity of the questions | | | | |
| --- | --- | --- | --- | --- | --- | --- | --- | --- | --- | --- | --- | --- | --- | --- | --- | --- |
| Item | Number  of experts | Number of  ratings | I-CVI | P_C_ | k* | Evalu  ation | Number  of ratings | I-CVI | Pc | k* | Evalu  ation | Number of  ratings | I-CVI | Pc | k* | Evalu  ation |
| 1 | 10 | 9 | 0.90 | 0.010 | 0.90 | **** | 9 | 0.90 | 0.010 | 0.90 | **** | 10 | 1.00 | 0.001 | 1.00 | **** |
| 2 | 10 | 9 | 0.90 | 0.010 | 0.90 | **** | 10 | 1.00 | 0.001 | 1.00 | **** | 10 | 1.00 | 0.001 | 1.00 | **** |
| 3 | 10 | 10 | 1.00 | 0.001 | 1.00 | **** | 10 | 1.00 | 0.001 | 1.00 | **** | 10 | 1.00 | 0.001 | 1.00 | **** |
| 4 | 10 | 9 | 0.90 | 0.010 | 0.90 | **** | 10 | 1.00 | 0.001 | 1.00 | **** | 8 | 0.80 | 0.044 | 0.79 | **** |

Table 4.2

Content validity of the dimension “Technological Pedagogical and Content Knowledge”

|  |  | Relevance of the questions | | | | | Clarity of the questions | | | | | Simplicity of the questions | | | | |
| --- | --- | --- | --- | --- | --- | --- | --- | --- | --- | --- | --- | --- | --- | --- | --- | --- |
| Item | Number  of experts | Number of  ratings | I-CVI | P_C_ | k* | Evalu  ation | Number  of ratings | I-CVI | Pc | k* | Evalu  ation | Number of  ratings | I-CVI | Pc | k* | Evalu  ation |
| 1 | 10 | 10 | 1.00 | 0.001 | 1.00 | **** | 8 | 0.80 | 0.044 | 0.79 | **** | 9 | 0.90 | 0.010 | 0.90 | **** |
| 2 | 10 | 10 | 1.00 | 0.001 | 1.00 | **** | 10 | 1.00 | 0.001 | 1.00 | **** | 10 | 1.00 | 0.001 | 1.00 | **** |
| 3 | 10 | 10 | 1.00 | 0.001 | 1.00 | **** | 9 | 0.90 | 0.010 | 0.90 | **** | 8 | 0.80 | 0.79 | 0.79 | **** |
| 4 | 10 | 10 | 1.00 | 0.001 | 1.00 | **** | 9 | 0.90 | 0.010 | 0.90 | **** | 10 | 1.00 | 0.001 | 1.00 | **** |
| 5 | 10 | 10 | 1.00 | 0.001 | 1.00 | **** | 9 | 0.90 | 0.010 | 0.90 | **** | 8 | 0.80 | 0.79 | 0.79 | **** |

Table 4.2

Content validity of the dimension “Behavioral Beliefs”

|  |  | Relevance of the questions | | | | | Clarity of the questions | | | | | Simplicity of the questions | | | | |
| --- | --- | --- | --- | --- | --- | --- | --- | --- | --- | --- | --- | --- | --- | --- | --- | --- |
| Item | Number  of experts | Number of  ratings | I-CVI | P_C_ | k* | Evalu  ation | Number  of ratings | I-CVI | Pc | k* | Evalu  ation | Number of  ratings | I-CVI | Pc | k* | Evalu  ation |
| 1 | 10 | 10 | 1.00 | 0.001 | 1.00 | **** | 10 | 1.00 | 0.001 | 1.00 | **** | 9 | 0.90 | 0.010 | 0.90 | **** |
| 2 | 10 | 9 | 0.90 | 0.010 | 0.90 | **** | 10 | 1.00 | 0.001 | 1.00 | **** | 9 | 0.90 | 0.010 | 0.90 | **** |
| 3 | 10 | 10 | 1.00 | 0.001 | 1.00 | **** | 10 | 1.00 | 0.001 | 1.00 | **** | 10 | 1.00 | 0.001 | 1.00 | **** |
| 4 | 10 | 9 | 0.90 | 0.010 | 0.90 | **** | 10 | 1.00 | 0.001 | 1.00 | **** | 10 | 1.00 | 0.001 | 1.00 | **** |
| 5 | 10 | 10 | 1.00 | 0.001 | 1.00 | **** | 10 | 1.00 | 0.001 | 1.00 | **** | 10 | 1.00 | 0.001 | 1.00 | **** |
| 6 | 10 | 10 | 1.00 | 0.001 | 1.00 | **** | 10 | 1.00 | 0.001 | 1.00 | **** | 10 | 1.00 | 0.001 | 1.00 | **** |
| 7 | 10 | 10 | 1.00 | 0.001 | 1.00 | **** | 9 | 0.90 | 0.010 | 0.90 | **** | 10 | 1.00 | 0.001 | 1.00 | **** |
| 8 | 10 | 9 | 0.90 | 0.010 | 0.90 | **** | 10 | 1.00 | 0.001 | 1.00 | **** | 10 | 1.00 | 0.001 | 1.00 | **** |

Table 4.2

Content validity of the dimension “Normative Beliefs”

|  |  | Relevance of the questions | | | | | Clarity of the questions | | | | | Simplicity of the questions | | | | |
| --- | --- | --- | --- | --- | --- | --- | --- | --- | --- | --- | --- | --- | --- | --- | --- | --- |
| Item | Number  of experts | Number of  Ratings | I-CVI | P_C_ | k* | Evalu  ation | Number  of ratings | I-CVI | Pc | k* | Evalu  ation | Number of  Ratings | I-CVI | Pc | k* | Evalu  ation |
| 1 | 10 | 10 | 1.00 | 0.001 | 1.00 | **** | 10 | 1.00 | 0.001 | 1.00 | **** | 10 | 1.00 | 0.001 | 1.00 | **** |
| 2 | 10 | 10 | 1.00 | 0.001 | 1.00 | **** | 10 | 1.00 | 0.001 | 1.00 | **** | 9 | 0.90 | 0.010 | 0.90 | **** |
| 3 | 10 | 8 | 0.80 | 0.044 | 0.79 | **** | 10 | 1.00 | 0.001 | 1.00 | **** | 10 | 1.00 | 0.001 | 1.00 | **** |
| 4 | 10 | 10 | 1.00 | 0.001 | 1.00 | **** | 10 | 1.00 | 0.001 | 1.00 | **** | 10 | 1.00 | 0.001 | 1.00 | **** |
| 5 | 10 | 10 | 1.00 | 0.001 | 1.00 | **** | 10 | 1.00 | 0.001 | 1.00 | **** | 8 | 0.80 | 0.044 | 0.79 | **** |

Table 4.2

Content validity of the dimension “Control Beliefs”

|  |  | Relevance of the questions | | | | | Clarity of the questions | | | | | Simplicity of the questions | | | | |
| --- | --- | --- | --- | --- | --- | --- | --- | --- | --- | --- | --- | --- | --- | --- | --- | --- |
| Item | Number  of experts | Number of  ratings | I-CVI | P_C_ | k* | Evalu  ation | Number  of ratings | I-CVI | Pc | k* | Evalu  ation | Number of  ratings | I-CVI | Pc | k* | Evalu  ation |
| 1 | 10 | 8 | 0.80 | 0.044 | 0.79 | **** | 10 | 1.00 | 0.001 | 1.00 | **** | 8 | 0.80 | 0.044 | 0.79 | **** |
| 2 | 10 | 10 | 1.00 | 0.001 | 1.00 | **** | 10 | 1.00 | 0.001 | 1.00 | **** | 10 | 1.00 | 0.001 | 1.00 | **** |
| 3 | 10 | 10 | 1.00 | 0.001 | 1.00 | **** | 10 | 1.00 | 0.001 | 1.00 | **** | 10 | 1.00 | 0.001 | 1.00 | **** |
| 4 | 10 | 10 | 1.00 | 0.001 | 1.00 | **** | 10 | 1.00 | 0.001 | 1.00 | **** | 10 | 1.00 | 0.001 | 1.00 | **** |
| 5 | 10 | 10 | 1.00 | 0.001 | 1.00 | **** | 10 | 1.00 | 0.001 | 1.00 | **** | 10 | 1.00 | 0.001 | 1.00 | **** |

Table 4.2

Content validity of the dimension “ICT Integration during Teaching Practices”

|  |  | Relevance of the questions | | | | | Clarity of the questions | | | | | Simplicity of the questions | | | | |
| --- | --- | --- | --- | --- | --- | --- | --- | --- | --- | --- | --- | --- | --- | --- | --- | --- |
| Item | Number  of experts | Number of  ratings | I-CVI | P_C_ | k* | Evalu  ation | Number  of ratings | I-CVI | Pc | k* | Evalu  ation | Number of  ratings | I-CVI | Pc | k* | Evalu  ation |
| 1 | 10 | 10 | 1.00 | 0.001 | 1.00 | **** | 10 | 1.00 | 0.001 | 1.00 | **** | 9 | 0.90 | 0.010 | 0.90 | **** |
| 2 | 10 | 10 | 1.00 | 0.001 | 1.00 | **** | 10 | 1.00 | 0.001 | 1.00 | **** | 10 | 1.00 | 0.001 | 1.00 | **** |
| 3 | 10 | 10 | 1.00 | 0.001 | 1.00 | **** | 10 | 1.00 | 0.001 | 1.00 | **** | 10 | 1.00 | 0.001 | 1.00 | **** |
| 4 | 10 | 10 | 1.00 | 0.001 | 1.00 | **** | 9 | 0.90 | 0.010 | 0.90 | **** | 8 | 0.80 | 0.044 | 0.79 | **** |
| 5 | 10 | 10 | 1.00 | 0.001 | 1.00 | **** | 10 | 1.00 | 0.001 | 1.00 | **** | 10 | 1.00 | 0.001 | 1.00 | **** |
| 6 | 10 | 10 | 1.00 | 0.001 | 1.00 | **** | 10 | 1.00 | 0.001 | 1.00 | **** | 10 | 1.00 | 0.001 | 1.00 | **** |
| 7 | 10 | 9 | 0.90 | 0.010 | 0.90 | **** | 9 | 0.90 | 0.010 | 0.90 | **** | 10 | 1.00 | 0.001 | 1.00 | **** |
| 8 | 10 | 10 | 1.00 | 0.001 | 1.00 | **** | 10 | 1.00 | 0.001 | 1.00 | **** | 10 | 1.00 | 0.001 | 1.00 | **** |
| 9 | 10 | 10 | 1.00 | 0.001 | 1.00 | **** | 9 | 0.90 | 0.010 | 0.90 | **** | 10 | 1.00 | 0.001 | 1.00 | **** |
| 10 | 10 | 8 | 0.80 | 0.044 | 0.79 | **** | 10 | 1.00 | 0.001 | 1.00 | **** | 10 | 1.00 | 0.001 | 1.00 | **** |
| 11 | 10 | 10 | 1.00 | 0.001 | 1.00 | **** | 9 | 0.90 | 0.010 | 0.90 | **** | 9 | 0.90 | 0.010 | 0.90 | **** |
| 12 | 10 | 10 | 1.00 | 0.001 | 1.00 | **** | 10 | 1.00 | 0.001 | 1.00 | **** | 9 | 0.90 | 0.010 | 0.90 | **** |
