# Supplementary material for: The dataset for validation of factors affecting pre-service teachers' use of ICT during teaching practices: Indonesian context
Source: Data Brief. 2019 Nov 26;28:104875. doi: 10.1016/j.dib.2019.104875 (PMC6911973; doi:10.1016/j.dib.2019.104875)
Supplement: Multimedia component 1 [file mmc1.zip › Questionnaire (English version).docx]

**Demographic Information**

**University**

1. Gender
a. Female
b. Male

2. Age range
a. 18-19
b. 20-21
c. >22

3. Major
a. Social science education
b. Science education
c. Language education

d. Pre-school or elementary teacher education

4. Semester
a. 4-5

b. 6-7

c. 8-9

5. How many ICT-related course have you attended?
a. 1
b. 2-3

c. >3

6. Are you currently enrolled or have you completed a practicum experience in a PK–6 classroom?
a. Yes
b. No

7. Do you want join FGD session discussing ICT integration during teaching practices?

a. Yes

b. No

If yes, provide email for invitation and certificate

_____________________________________

Please tick in provided column

1= Very disagree

2= Disagree

3= Neutral

4= Agree

5= Very agree

**A. Technological pedagogical and content knowledge (TPACK)**

**Technological knowledge (TK)**

| No | Statement | 1 | 2 | 3 | 4 | 5 |
| --- | --- | --- | --- | --- | --- | --- |
|  | I have the technical skills to use ICT. |  |  |  |  |  |
|  | I can learn ICT easily. |  |  |  |  |  |
|  | I know how to solve my own ICT technical problems. |  |  |  |  |  |

**Content knowledge (CK)**

| No | Statement | 1 | 2 | 3 | 4 | 5 |
| --- | --- | --- | --- | --- | --- | --- |
|  | I have sufficient knowledge about my content area. |  |  |  |  |  |
|  | I can think about the subject matter like an expert who specializes in my content area. |  |  |  |  |  |
|  | I have various ways and strategies of developing my understanding of my content area. |  |  |  |  |  |

**Pedagogical knowledge (PK)**

| No | Statement | 1 | 2 | 3 | 4 | 5 |
| --- | --- | --- | --- | --- | --- | --- |
|  | I know how to assess student performance in a classroom. |  |  |  |  |  |
|  | I can adapt my teaching based upon what students currently understand or do not understand. |  |  |  |  |  |
|  | I can adapt my teaching style to diﬀerent learners. |  |  |  |  |  |
|  | I can assess student learning in multiple ways. |  |  |  |  |  |
|  | I can use a wide range of teaching approaches in a classroom setting. |  |  |  |  |  |
|  | I am familiar with common student understandings and misconceptions. |  |  |  |  |  |
|  | I know how to organize and maintain classroom management. |  |  |  |  |  |

**Pedagogical content knowledge (PCK)**

| No | Statement | 1 | 2 | 3 | 4 | 5 |
| --- | --- | --- | --- | --- | --- | --- |
|  | Without ICT, I can select eﬀective teaching approaches to guide student thinking and learning in my content area. |  |  |  |  |  |
|  | Without using ICT, I can address the common misconceptions my students have for my content area |  |  |  |  |  |
|  | Without using ICT, I can help my students understand the content knowledge through various ways |  |  |  |  |  |

**Technological content knowledge (TCK)**

| No | Statement | 1 | 2 | 3 | 4 | 5 |
| --- | --- | --- | --- | --- | --- | --- |
|  | I know about ICT that I can use for understanding and doing my content area. |  |  |  |  |  |
|  | I can use appropriate ICT to represent the content of my teaching |  |  |  |  |  |
|  | I can use the ICT that are created specifically for content area. |  |  |  |  |  |

**Technological pedagogical knowledge (TPK)**

| No | Statement | 1 | 2 | 3 | 4 | 5 |
| --- | --- | --- | --- | --- | --- | --- |
|  | I can choose ICT that enhance the teaching approaches for a lesson. |  |  |  |  |  |
|  | My teacher education program has caused me to think more deeply about how ICT could inﬂuence the teaching approaches I use in my classroom. |  |  |  |  |  |
|  | I am thinking critically about how to use ICT in my classroom. |  |  |  |  |  |
|  | I can adapt the use of the ICT that I am learning about to diﬀerent teaching activities. |  |  |  |  |  |

**Technological pedagogical and content knowledge (TPACK)**

| No | Statement | 1 | 2 | 3 | 4 | 5 |
| --- | --- | --- | --- | --- | --- | --- |
|  | I can teach lessons that appropriately combine my content area, ICT, and teaching approaches. |  |  |  |  |  |
|  | I can select ICT to use in my classroom that enhances what I teach, how I teach, and what students learn. |  |  |  |  |  |
|  | I can use strategies that combine content, ICT, and teaching approaches that I learned about in my coursework in my classroom. |  |  |  |  |  |
|  | I can provide leadership in helping others to coordinate the use of content, ICT, and teaching approaches at my school and/or district. |  |  |  |  |  |
|  | I can choose ICT that enhance the content for a lesson. |  |  |  |  |  |

**B. Beliefs on ICT**

**Behavioral beliefs (BB)**

| No | Statement | 1 | 2 | 3 | 4 | 5 |
| --- | --- | --- | --- | --- | --- | --- |
|  | I think that using ICT provides possibilities for enhancing the quality of learning |  |  |  |  |  |
|  | I think that ICT integration improves engagement with content and students |  |  |  |  |  |
|  | Integrating ICT during teaching practices is relevant with the 21^st^ century teaching |  |  |  |  |  |
|  | I think that ICT integration enriches learning experience through innovative tools |  |  |  |  |  |
|  | I think that ICT integration helps facilitate understanding of material/concepts |  |  |  |  |  |
|  | I feel that ICT is easy to use |  |  |  |  |  |
|  | The ICT integration caters to the needs of different learning styles |  |  |  |  |  |
|  | I think that using ICT improves students’ evaluation in their learning |  |  |  |  |  |

| No | Statement | 1 | 2 | 3 | 4 | 5 |
| --- | --- | --- | --- | --- | --- | --- |
|  | My colleagues expect me to use ICT for teaching. |  |  |  |  |  |
|  | The principal of my school expect me to use ICT for teaching. |  |  |  |  |  |
|  | My students assume that I can use ICT for teaching. |  |  |  |  |  |
|  | Parents of my students expect me to use ICT for teaching. |  |  |  |  |  |
|  | My professor expects me to integrate ICT for teaching. |  |  |  |  |  |

**Normative beliefs (NB)**

**Control beliefs (CB)**

| No | Statement | 1 | 2 | 3 | 4 | 5 |
| --- | --- | --- | --- | --- | --- | --- |
|  | I think that the advantages of using ICT outweigh the disadvantages. |  |  |  |  |  |
|  | I have high self-efficacy in personal use in teaching using ICT. |  |  |  |  |  |
|  | I think access to learning outside the classroom supports the ICT use. |  |  |  |  |  |
|  | I think that access to ICT is not a problem in ICT integration. |  |  |  |  |  |
|  | I think supporting human resources is not an issue for ICT integration. |  |  |  |  |  |

**C. Pre-Service teachers’ use of ICT during teaching practices (UICT)**

| No | Statement | 1 | 2 | 3 | 4 | 5 |
| --- | --- | --- | --- | --- | --- | --- |
|  | I use search engines (e.g. Google, Yahoo, etc.) to look for information on the Internet. |  |  |  |  |  |
|  | I do assessment using ICT. |  |  |  |  |  |
|  | I create instructional materials (e.g. handouts, tests, etc.) using ICT. |  |  |  |  |  |
|  | I create lesson plans using ICT. |  |  |  |  |  |
|  | I use data-show projectors to present subject matter. |  |  |  |  |  |
|  | I use presentation programs (e.g. PowerPoint, etc.) to present subject matter. |  |  |  |  |  |
|  | I prepare visual effects to enhance learning concepts through ICT. |  |  |  |  |  |
|  | I prepare sound effects to enhance learning concepts through ICT. |  |  |  |  |  |
|  | I use the Internet e.g. social media, wiki, etc. for my instruction. |  |  |  |  |  |
|  | I use ICT for simulation. |  |  |  |  |  |
|  | I use computers or smartphones application in my instructional activities. |  |  |  |  |  |
|  | I use ICT to communicate with my students. |  |  |  |  |  |
