# Supplementary material for: The dataset for validation of factors affecting pre-service teachers' use of ICT during teaching practices: Indonesian context
Source: Data Brief. 2019 Nov 26;28:104875. doi: 10.1016/j.dib.2019.104875 (PMC6911973; doi:10.1016/j.dib.2019.104875)
Supplement: Multimedia component 1 [file mmc1.zip › Questionnaire (Indonesian version).docx]

**Informasi Demografi**

**Universitas**

1. Jenis kelamin:

1. Perempuan
2. Laki-laki

2. Rentang umur:

a. 18-19
b. 20-21
c. >22

4. Jurusan:
 a. Pendidikan Ilmu sosial

b. Pendidikan Ilmu sains

c. Pendidikan Bahasa

d. Pendidikan guru sekolah dasar atau taman kanak-kanak

5. Semester:

a. 4-5

b. 6-7

c. 8-9

7. Berapa mata kuliah TIK (yang berhubungan) yang telah kamu selesaikan?

a. 1
b. 2-3

c. >3

8. Apakah kamu telah melaksanakan PPL atau sedang melaksanakan PPL?

a. Ya
b. Tidak

9. Apakah kamu ingin ikut sesi diskusi membahas tentang integrasi TIK dalam pengajaran PPL?

a. Ya

b. Tidak

Jika Ya, cantumkan email kamu untuk kami kirimkan undangan dan sertifikat

_____________________________________

Keterangan angka

Conteng ( ) dikolom yang telah disediakan

1= Sangat Tidak Setuju

2= Tidak Setuju

3= Netral

4= Setuju

5= Sangat Setuju

**A. TPACK**

**TK**

| No | Pernyataan | 1 | 2 | 3 | 4 | 5 |
| --- | --- | --- | --- | --- | --- | --- |
|  | *Saya memiliki keterampilan teknis untuk menggunakan TIK*. |  |  |  |  |  |
|  | *Saya bisa dengan mudah belajar TIK* |  |  |  |  |  |
|  | *Saya tahu cara menyelesaikan masalah teknis TIK yang saya hadapi* |  |  |  |  |  |

**CK**

| No | Pernyataan | 1 | 2 | 3 | 4 | 5 |
| --- | --- | --- | --- | --- | --- | --- |
|  | *Saya punya cukup pengetahuan tentang materi yang saya ajarkan* |  |  |  |  |  |
|  | *Saya dapat berpikir tentang materi pelajaran seperti seorang ahli yang mengkhususkan diri dalam materi yang saya ajarkan* |  |  |  |  |  |
|  | *Saya memiliki berbagai cara dan strategi untuk mengembangkan pemahaman saya tentang materi yang saya ajarkan* |  |  |  |  |  |

**PK**

| No | Pernyataan | 1 | 2 | 3 | 4 | 5 |
| --- | --- | --- | --- | --- | --- | --- |
|  | S*aya tahu bagaimana menilai kinerja siswa di kelas.* |  |  |  |  |  |
|  | *Saya dapat menyesuaikan pengajaran saya berdasarkan apa yang saat ini siswa pahami dan tidak mereka pahami.* |  |  |  |  |  |
|  | *Saya dapat menyesuaikan gaya mengajar saya dengan siswa yang berbeda karakter.* |  |  |  |  |  |
|  | *Saya dapat mengevaluasi hasil belajar siswa dengan berbagai cara.* |  |  |  |  |  |
|  | *Dalam mengajar, saya dapat menggunakan berbagai pendekatan pengajaran.* |  |  |  |  |  |
|  | *Saya akrab dengan pemahaman dan ketidakpahaman siswa* |  |  |  |  |  |
|  | *Saya tahu cara mengatur dan mengelola kelas* |  |  |  |  |  |

**PCK**

| No | Pernyataan | 1 | 2 | 3 | 4 | 5 |
| --- | --- | --- | --- | --- | --- | --- |
|  | *Tanpa menggunakan TIK, saya dapat memilih pendekatan pengajaran yang efektif untuk mengembangkan pembelajaran dan pemikiran siswa akan materi yang saya ajarkan.* |  |  |  |  |  |
|  | *Tanpa menggunakan TIK, saya bias menjelaskan kesalahpahaman umum siswa akan materi yang saya ajarkan* |  |  |  |  |  |
|  | *Tanpa menggunakan TIK, saya bias membantu siswa memahami materi dengan belpagai cara* |  |  |  |  |  |

**TCK**

| No | Pernyataan | 1 | 2 | 3 | 4 | 5 |
| --- | --- | --- | --- | --- | --- | --- |
|  | *Saya memahami TIK yang dapat saya gunakan untuk memahami dan mengerjakan materi yang saya ajarkan* |  |  |  |  |  |
|  | *Saya biasa menggunakan TIK yang tepat untuk mwakili materi yang saya ajarkan.* |  |  |  |  |  |
|  | *Saya bisa menggunakan TIK yang diciptakan khusus untuk materi yang saya ajarkan* |  |  |  |  |  |

**TPK**

| No | Pernyataan | 1 | 2 | 3 | 4 | 5 |
| --- | --- | --- | --- | --- | --- | --- |
|  | *Saya dapat memilih TIK dalam meningkatkan pendekatan pengajaran untuk sebuah mata pelajaran.* |  |  |  |  |  |
|  | *Program pendidikan di mana saya belajar membuat saya berpikir lebih baik tentang bagaimana TIK dapat memengaruhi pendekatan pengajaran yang saya gunakan di kelas.* |  |  |  |  |  |
|  | *Saya berpikir kritis tentang bagaimana menggunakan TIK di kelas saya.* |  |  |  |  |  |
|  | *Saya dapat menyesuaikan penggunaan TIK yang saya pelajari dalam kegiatan pengajaran di kelas berbeda.* |  |  |  |  |  |

**TPACK**

| No | Pernyataan | 1 | 2 | 3 | 4 | 5 |
| --- | --- | --- | --- | --- | --- | --- |
|  | *Saya dapat mengajarkan mata pelajaran secara tepat dengan menggabungkan materi yang saya ajarkan, TIK dan pendekatan pengajaran.* |  |  |  |  |  |
|  | *Saya dapat memilih TIK dalam rangka digunakan di kelas untuk meningkatkan apa yang saya ajarkan, bagaimana saya mengajar, dan apa yang dipelajari siswa.* |  |  |  |  |  |
|  | *Saya dapat menggunakan strategi yang menggabungkan materi yang saya ajarkan, TIK, dan pendekatan pengajaran yang saya pelajari di universitas* |  |  |  |  |  |
|  | *Saya dapat memimpin dalam membantu orang lain untuk mengkoordinasikan materi yang saya ajarkan, TIK, dan pendekatan pengajaran di sekolah tempat saya mengajar.* |  |  |  |  |  |
|  | *Saya dapat memilih TIK untuk meningkatkan pemahaman akan materi yang saya ajarkan dan pendekatan pengajaran*. |  |  |  |  |  |

**B. Beliefs on ICT**

**Behavioral beliefs**

| No | Pernyataan | 1 | 2 | 3 | 4 | 5 |
| --- | --- | --- | --- | --- | --- | --- |
|  | *Saya berpendapat bahwa menggunakan TIK memberikan kemungkinan untuk meningkatkan kualitas pembelajaran.* |  |  |  |  |  |
|  | *Saya berpendapat bahwa integrasi TIK meningkatkan keterlibatan dengan materi dan siswa.* |  |  |  |  |  |
|  | *Integrasi TIK selama praktik mengajar relevan dengan pengajaran abad ke-21.* |  |  |  |  |  |
|  | *Saya berpendapat bahwa integrasi TIK memperkaya pengalaman belajar melalui alat-alat yang inovatif.* |  |  |  |  |  |
|  | *Saya berpendapat bahwa integrasi TIK membantu memfasilitasi pemahaman materi / konsep.* |  |  |  |  |  |
|  | *Saya rasa TIK mudah digunakan* |  |  |  |  |  |
|  | *Integrasi TIK melayani kebutuhan gaya belajar yang berbeda.* |  |  |  |  |  |
|  | *Saya berpendapat bahwa menggunakan TIK meningkatkan evaluasi siswa dalam pembelajaran mereka.* |  |  |  |  |  |

| No | Pernyataan | 1 | 2 | 3 | 4 | 5 |
| --- | --- | --- | --- | --- | --- | --- |
|  | *Rekan-rekan saya mengharapkan saya menggunakan TIK untuk mengajar.* |  |  |  |  |  |
|  | *Kepala sekolah saya mengharapkan saya menggunakan TIK untuk pengajaran.* |  |  |  |  |  |
|  | *Murid-murid saya berasumsi bahwa saya dapat menggunakan TIK untuk mengajar.* |  |  |  |  |  |
|  | *Orang tua siswa mengharapkan saya menggunakan TIK untuk mengajar.* |  |  |  |  |  |
|  | *Dosen saya mengharapkan saya untuk mengintegrasikan TIK untuk mengajar.* |  |  |  |  |  |

**Normative beliefs**

**Control beliefs**

| No | Pernyataan | 1 | 2 | 3 | 4 | 5 |
| --- | --- | --- | --- | --- | --- | --- |
|  | *Saya berpendapat bahwa manfaat menggunakan TIK lebih besar daripada kerugiannya.* |  |  |  |  |  |
|  | *Saya memiliki keyakinan diri yang tinggi dalam pengajaran menggunakan TIK.* |  |  |  |  |  |
|  | *Saya berpendapat bahwa akses untuk belajar di luar kelas mendukung penggunaan TIK.* |  |  |  |  |  |
|  | *Saya berpendapat bahwa akses ke TIK bukan masalah dalam integrasi TIK.* |  |  |  |  |  |
|  | *Saya berpendapat bahwa SDM yang medukung bukan masalah dalam integrasi TIK.* |  |  |  |  |  |

**C. PRE-SERVICE TEACHERS’ USE OF ICT DURING TEACHING PRACTICES**

| No | Pernyataan | 1 | 2 | 3 | 4 | 5 |
| --- | --- | --- | --- | --- | --- | --- |
|  | *Saya menggunakan mesin pencari internet (misalnya Google, Yahoo, dll.) dalam menyiapkan materi dan pelaksanaan pembelajaran.* |  |  |  |  |  |
|  | *Saya melakukan penilaian menggunakan TIK* |  |  |  |  |  |
|  | *Saya membuat bahan ajar (misalnya handout, tes, dll) menggunakan TIK* |  |  |  |  |  |
|  | *Saya membuat RPP menggunakan TIK* |  |  |  |  |  |
|  | *Saya menggunakan proyektor untuk menyajikan materi pelajaran* |  |  |  |  |  |
|  | *Saya menggunakan program presentasi (misalnya PowerPoint, dll) untuk menyajikan materi pelajaran* |  |  |  |  |  |
|  | *Saya menyiapkan efek visual untuk meningkatkan konsep pembelajaran melalui TIK* |  |  |  |  |  |
|  | *Saya menyiapkan efek audio untuk meningkatkan konsep pembelajaran melalui TIK* |  |  |  |  |  |
|  | *Saya menggunakan internet e.g media social, wiki, dll untuk dalam kegiatan pembelajaran* |  |  |  |  |  |
|  | *Saya menggunakan TIK untuk simulasi pembelajaran.* |  |  |  |  |  |
|  | *Saya menggunakan applikasi komputer atau smartphone dalam kegiatan pembelajaran* |  |  |  |  |  |
|  | *Saya menggunakan TIK dalam komunikasi dengan siswa* |  |  |  |  |  |
